# Supplementary material for: Safe abortion service utilization and associated factors among insecurely housed women who experienced abortion in southwest Ethiopia, 2021: A community-based cross-sectional study
Source: PLoS One. 2022 Aug 18;17(8):e0272939. doi: 10.1371/journal.pone.0272939 (PMC9387822; doi:10.1371/journal.pone.0272939)
Supplement: S1 File — (DOCX) [file pone.0272939.s001.docx]

**THIS QUESTIONNAIRE IS PREPARED FOR COLLECTING INFORMATION ON UTILIZATION OF SAFE ABORTION SERVICE AND ASSOCIATED FACTORS AMONG INSECURELY HOUSED WOMEN IN SOUTHWEST ETHIOPIA**

Questionnaire code: _______

Good morning/ good afternoon/ good evening.

My name is_________________________

We are conducting research entitled with utilization of safe abortion service and associated factors among Insecurely Housed women.

I promise to keep confidentiality of your response. Your name is not included in any data processing in the research and will never be used in connection with any of the information you give. You are kindly requested to answer every question and you may stop responding the questions at any time you want to. However, your honest answers to these questions will help for better understanding of this study. There can be more than one answer as given on the alternative choices or opinions. The total time needed for responding this questionnaire is about thirty minutes. So, I kindly request you to participate in genuinely answering the interview.

Are you voluntary to participate? Yes ----- No -----

If yes put your Signature -------------- date -------/ -------- /------------

If No thank you

1. **Socio demographic questions**
2. Age: _______
3. Residence
4. Jimma
5. Bonga
6. Mizan-aman
7. Insecurely housed by type
8. I depend on the street for my life and return home at night (of- the-street).
9. I depend on the street for my life and sleep on the street (On-the-street).
10. Religion:
11. Orthodox
12. Muslim
13. Protestant
14. Catholic
15. Other (specify) _____________
16. Marital status:
17. Single
18. Married
19. Widowed
20. Divorced
21. Ethnic group
22. Oromo
23. Dawro
24. Kaffa
25. Amhara
26. Yem
27. Others (specify) ______________
28. Educational status
29. Never attend school
30. Primary school
31. High school
32. Collage and above
33. Average income ____________________
34. Source of income ___________________
35. **Pregnancy and abortion experience**
36. Have you ever had history of pregnancy?
37. Yes
38. No
39. If yes, how many? ______
40. Is your recent pregnancy wanted?
41. Yes
42. No
43. Have you ever know the one who has abortion experience?
44. Yes
45. No
46. What about you? Have you ever had abortion experience?
47. Yes
48. No
49. If yes, what type of abortion is it?
50. Induced
51. Spontaneous
52. **Questions to assess knowledge**
53. Do you know that safe abortion is provided in health facilities?
54. Yes
55. No
56. Have you ever heard about the current abortion law of Ethiopian?
57. Yes
58. No
59. What do you know about abortion law in Ethiopia?
60. Abortion is legal in Ethiopia and provided on request
61. Abortion is illegal in Ethiopia
62. Abortion is legal for some selected reasons
63. Don’t know

If your answer for Q19 is 3, in what do you think are prerequisites for abortion?

1. Fetal malformation
2. Yes
3. No
4. Continuing pregnancy endanger woman’s life
5. Yes
6. No
7. Pregnancy at age below 18 years
8. Yes
9. No
10. Pregnancy results from rape or incents
11. Yes
12. No
13. If mother can’t bring up her child due to physical or mental illness
14. Yes
15. No
16. If mother can’t bring up her child due extreme poverty
17. Yes
18. No
19. **Individual and health system related questions**
20. Have you ever faced stigma/ discrimination from health care providers?
21. Yes
22. No
23. Do you think that you might face stigma/ discrimination from health care provider?
24. Yes
25. No
26. Have you ever had pre-abortion counseling at health facility?
27. Yes
28. No
29. Have you ever counseled to continue pregnancy at health facility
30. Yes
31. No
32. Have you ever waited prolonged time to receive abortion service at health facility?
33. Yes
34. No
35. How do you rate the cost abortion in health facilities?
36. I think it is Free
37. I think it is Affordable
38. I think it is unaffordable
39. **Questions to assess safe abortion service utilization**
40. What is your reason to induce abortion
41. Unwanted pregnancy
42. Economical reason
43. Medical condition
44. Other (specify)
45. Where did abortion takes place
46. Health institution
47. Outside health institution
48. If in health institution, which one?
49. Health center
50. Private clinics
51. Public hospitals
52. Private hospitals
53. If outside health institution, where?
54. Self induced
55. In informal setting by traditional provider
56. Others (specify)
