# Supplementary material for: Safe abortion service utilization and associated factors among insecurely housed women who experienced abortion in southwest Ethiopia, 2021: A community-based cross-sectional study
Source: PLoS One. 2022 Aug 18;17(8):e0272939. doi: 10.1371/journal.pone.0272939 (PMC9387822; doi:10.1371/journal.pone.0272939)
Supplement: Attachment — Submitted filename: Thank you for the opportunity to review this manuscript.docx [file pone.0272939.s003.docx]

Thank you for the opportunity to review this manuscript. The manuscript reports the findings of a study of safe-abortion services use among reproductive-age street-involved insecurely housed women in Southwest Ethiopia. The authors explored the prevalence and predictors of accessing safe-abortion services by insecurely housed women by surveying a convenience sample of 124 women and conducting a multivariant logistic regression with use of safe abortion services (yes/no) as the binary outcome variable. The surveyed insecurely housed women were found to be significantly less likely to access safe abortion services than the general population in Ethiopia, while income, knowledge of available safe-abortion services and perceptions about the cost of such services being the significant predictors of not accessing safe-abortions.

The manuscript reports the findings of an important and interesting study on an extremely marginalized and understudied population with important implications for our understanding of reproductive health behaviors and the barriers marginalized populations navigate as they try to access reproductive health services. Most importantly the findings show that the choice is between safe and unsafe abortion services, and that insecurely housed women on the margins of the Ethiopian society, are not utilizing safe and freely available abortion services due to lack of knowledge and misconceptions about costs.

However, the manuscript requires some significant revisions before it will be ready for publication.

1. The manuscript requires careful language editing. At times the inconsistent language, grammatical errors, and some (not many) typos prevents from fully understanding the authors’ intentions.
2. The terminology used is not consistent with current conventions of writing about people experiencing insecure housing and homelessness. Though the authors refer to their sample as homeless women, at least 26% of them were street-involved in that they were panhandling (the authors term this ‘begging’) however they slept indoors. The authors (p.13) differentiate between on-the street and off-the street women with ‘off-the-street” women defined as those sleeping in the street. This may be a typo and the authors meant that the other way around, in which case 70% of the sample were street-involved but insecurely housed rather than homeless. I would suggest terming the sample insecurely housed women rather than homeless women. The terms ‘street girls’ and ‘street women’ (p.10) should be replaced with street-involved
3. The quantitative analysis requires significant revisions. Rather than a proper statistical power analysis the authors used a thumb rule (10 participants – not ‘samples’ as the authors write – per predictor variable) this should be replaced by a post-hoc power analysis reporting the power provided by the 124 participants when running a multivariate logistic regression with 3 predictors (the final model). Reliability of measures is not well reported, Cronbach’s alphas for each scale used should be reported both for the sample and from previous validation studies if available as well as some details about who developed these measures and why these were chosen. The authors state (p.12) they have committed some questions due to low reliability score – the full details of the process need to be provided including which questions were omitted, which scale thy belonged to originally and what were the scores. The process by which the multivariate logistic regression model was developed is not well described, how were the original predictors chosen and how were some of them omitted and the rest retained? The Hosmer-Lemeshow test of goodness of fit reported is usually not recommended due to low reliability. Providing a more fulsome description of the logistic regression conducted and the model fit indices is needed.
4. It is not clear why knowledge about safe-abortion services and the misconception about their cost are distinct factors – surely thinking a free service is unaffordable constitute an example for lack of knowledge – a better explanation as to why this were deemed distinct is needed.
5. The association between higher daily income and access to safe abortion services found is more perplexing than the discussion accounts for. Given the services are provided free of charge, is higher daily income a proxy for some other characteristic (e.g., higher executive functioning; greater autonomy in decision making)?

In sum I wish to reiterate that the manuscript is interesting and the study highly important and I hope some of my suggestions will prove helpful in revising this manuscript and resubmitting for publication.
