# Supplementary material for: Safe abortion service utilization and associated factors among insecurely housed women who experienced abortion in southwest Ethiopia, 2021: A community-based cross-sectional study
Source: PLoS One. 2022 Aug 18;17(8):e0272939. doi: 10.1371/journal.pone.0272939 (PMC9387822; doi:10.1371/journal.pone.0272939)
Supplement: Attachment — Submitted filename: Response to Reviewers.docx [file pone.0272939.s004.docx]

**AUTHORS’ RESPONSE TO ACADEMIC EDITOR AND REVIEWERS**

1. **Author’s Response To Editor**

**Dear: Lorena Verduci(Staff Editor)**

We thank you for a thorough reading and constructive comments and suggestions on our manuscript and for the opportunity to revise and resubmit. We are pleased to submit the revised research article “**Safe Abortion Service Utilization and Associated Factors among Homeless Women Who Experienced Abortion in Southwest Ethiopia, 2021: A Community-Based Cross-Sectional Study**” for your consideration in the special collection of Plos One. On the following pages, you will find our responses to the questions raised from your side and two reviewers' comments. On behalf of my co-authors, I thank you for your consideration of this resubmission. We appreciate your time and look forward to your response.

Sincerely,

Aklilu Habte(MPH)(corresponding author)

[**aklilihabte57@gmail.com**](mailto:aklilihabte57@gmail.com)

**Dear Dr. Habte,**

Dear Dr. Habte,

Thank you for submitting your manuscript to PLOS ONE. After careful consideration, we feel that it has merit but does not fully meet PLOS ONE’s publication criteria as it currently stands. Therefore, we invite you to submit a revised version of the manuscript that addresses the points raised during the review process.

Please note that we have only been able to secure a single reviewer to assess your manuscript. We are issuing a decision on your manuscript at this point to prevent further delays in the evaluation of your manuscript. Please be aware that the editor who handles your revised manuscript might find it necessary to invite additional reviewers to assess this work once the revised manuscript is submitted. However, we will aim to proceed on the basis of this single review if possible.

The manuscript has been evaluated by one reviewer, and his comments are available below.

The reviewer has raised a number of concerns. He requests improvements to the reporting of methodological aspects of the study (such as changes to the terminology used) revisions to the statistical analyses and to the language used.

Could you please carefully revise the manuscript to address all comments raised?

**Response:**Thank you for providing us with the opportunity to revise our work by incorporating all of the valuable comments and suggestions made by the reviewers. We are also grateful for the reviewers' insightful comments and suggestions, which we received all of them were beneficial for further improving the manuscript. In the "Response to Reviewers" section below, we endeavored to answer all constructive remarks and ideas raised by reviewers.

**Journal Requirements:**

When submitting your revision, we need you to address these additional requirements.

Comment1:Please ensure that your manuscript meets PLOS ONE's style requirements, including those for file naming. The PLOS ONE style templates can be found at

<https://journals.plos.org/plosone/s/file?id=wjVg/PLOSOne_formatting_sample_main_body.pdf> and

<https://journals.plos.org/plosone/s/file?id=ba62/PLOSOne_formatting_sample_title_authors_affiliations.pdf>

**Response:** Thank you for your suggestion. We have submitted the manuscript as per the journal requirement.

**Comment2:**We suggest you thoroughly copyedit your manuscript for language usage, spelling, and grammar. If you do not know anyone who can help you do this, you may wish to consider employing a professional scientific editing service. "Upon re-submitting your revised manuscript, please upload your study’s minimal underlying data set as either Supporting Information files or to a stable, public repository and include the relevant URLs, DOIs, or accession numbers within your revised cover letter. For a list of acceptable repositories, please see http://journals.plos.org/plosone/s/data-availability#loc-recommended-repositories. Any potentially identifying patient information must be fully anonymized.

**Response:**We appreciate your insightful comments and suggestions, which remind us to include the supporting information that we overlooked in the initial submission. We incorporate the minimal underlying data set that we used for the study's findings, per your suggestion.Accordingly, we incorporate both the “minimal data set” and “data collection tool” in the“supportive information” section of the “Revised manuscript with track changes” as “**S1-Data set**” and “**S1-Tool”**, Line 365-367, Page14, respectively.

**Comment3:**We suggest you thoroughly copyedit your manuscript for language usage, spelling, and grammar. If you do not know anyone who can help you do this, you may wish to consider employing a professional scientific editing service.

**Response:**Thank you very much for all of your thoughtful comments and recommendations, as well as your kind words, which have helped us to refine our work in its final form for the readers. In terms of language proficiency, we contacted the professional language editors who were working in the Department of Language and Literature at our university and they endeavored to address the issues. All the possible modifications and amendments were highlighted throughout the “Revised manuscript with Track Changes”

_____________________________END________________________________________

THANK YOU!!!

**AUTHORS’ RESPONSE TO REVIEWERS**

**Dear: Reviewers (Jonathan Alschech)**

We thank you for a thorough reading and constructive comments and suggestions on our manuscript and for the opportunity to revise and resubmit. We are pleased to submit the improved research article **“Safe Abortion Service Utilization and Associated Factors among Homeless Women Who Experienced Abortion in Southwest Ethiopia, 2021: A Community-Based Cross-Sectional Study”** for your consideration in the special collection of Plos One. On the following pages, you will find our response to the reviewers’ comments. On behalf of my co-authors, I thank you for your consideration of this resubmission. We appreciate your time and look forward to our responses to the comments and suggestions raised from your side as follows

Sincerely,

Aklilu Habte. (MPH in Reproductive Health)(corresponding author)

[**aklilihabte57@gmail.com**](mailto:aklilihabte57@gmail.com)

**Authors’ Response to Reviewer**#**1(Jonathan Alschech)**

**General comments**

Thank you for the opportunity to review this manuscript. The manuscript reports the findings of a study of safe-abortion services use among reproductive-age street-involved insecurely housed women in Southwest Ethiopia. The authors explored the prevalence and predictors of accessing safe-abortion services by insecurely housed women by surveying a convenience sample of 124 women and conducting a multivariant logistic regression with use of safe abortion services (yes/no) as the binary outcome variable. The surveyed insecurely housed women were found to be significantly less likely to access safe abortion services than the general population in Ethiopia, while income, knowledge of available safe-abortion services and perceptions about the cost of such services being the significant predictors of not accessing safe-abortions.

The manuscript reports the findings of an important and interesting study on an extremely marginalized and understudied population with important implications for our understanding of reproductive health behaviors and the barriers marginalized populations navigate as they try to access reproductive health services. Most importantly the findings show that the choice is between safe and unsafe abortion services, and that insecurely housed women on the margins of the Ethiopian society, are not utilizing safe and freely available abortion services due to lack of knowledge and misconceptions about costs.

However, the manuscript requires some significant revisions before it will be ready for publication.

**Response:** Thank you very much for taking the time to review our work and for your positive feedback. We got all your constructive comments and suggestions as valuable input for the readability of our work and we have tried to respond as follows. We give due emphasis to those grammatical and Punctuation errors by involving experts in language and literature and we have tried to correct them in the revised version of the manuscript.

**Specific comments**

**Comment1:**The manuscript requires careful language editing. At times the inconsistent language, grammatical errors, and some (not many) typos prevents from fully understanding the authors’ intentions.

**Response:** Thank you very much for all of your thoughtful comments and recommendations, as well as your kind words, which have helped us to refine our work in its final form for the readers. In terms of language proficiency, we contacted the professional language editors who were working in the Department of Language and Literature at our university and they endeavored to address the issues. All the possible modifications and amendments were highlighted throughout the “Revised manuscript with Track Changes”

**Comment2:**The terminology used is not consistent with current conventions of writing about people experiencing insecure housing and homelessness. Though the authors refer to their sample as homeless women, at least 26% of them were street-involved in that they were panhandling (the authors term this ‘begging’) however they slept indoors. The authors (p.13) differentiate between on-the street and off-the street women with ‘off-the-street” women defined as those sleeping in the street. This may be a typo and the authors meant that the other way around, in which case 70% of the sample were street-involved but insecurely housed rather than homeless. I would suggest terming the sample insecurely housed women rather than homeless women. The terms ‘street girls’ and ‘street women’ (p.10) should be replaced with street-involved

**Response:** Your insightful and well-articulated comments and suggestions are greatly appreciated. We accept your comments in their entirety and have corrected them accordingly. Throughout the manuscript, we replaced the term "homeless women" with "insecurely housed women" and the changes were highlighted. We apologize for any confusion caused by the definitions of on-the-street and off-the-street women, which was a typo error. We have now corrected the statements as per their standard definitions, and the corrected versions have been highlighted in the “Measurement of variables of the study” section of the “Revised manuscript with Track Changes”, Page7, Line 184-187.

**Comment3:**The quantitative analysis requires significant revisions. Rather than a proper statistical power analysis, the authors used a thumb rule (10 participants – not ‘samples’.

**Response:** Thank you for your thorough review; we have changed and highlighted it in the. “Sample size determination” section of the “Revised manuscript with Track Changes”, Page5, Line 142.

**Comment4:** as the authors write – per predictor variable) this should be replaced by a Post-hoc power analysis reporting the power provided by the 124 participants when running a multivariate logistic regression with 3 predictors (the final model).

**Response:** We value you reminding us to report the power analysis technique, which we have done but did not report. As per your suggestion, we have added the results of a Post-hoc power analysis in the “Data analysis” section of the “Revised manuscript with Track Changes”, Page7, Line 207-213.

**Comment5:** Reliability of measures is not well reported, Cronbach’s alphas for each scale used should be reported both for the sample and from previous validation studies if available..

**Response:** thank you for asking to incorporate the way how we assessed the reliability of the questionnaire. of course, conducted the reliability(internal consistency between the items of knowledge and practice assessment questions and the corresponding Cronbach’s alphas were 0.83 and 0.87, respectively. The internal consistency composite score was 0.84, which indicates that 84% of the variance in the scores was found to be reliable and deemed to be good. As per your recommendation, we have added it to the “Data quality management” section of the “Revised manuscript with Track Changes”, Page6, Line 167-172.

**Comment6:** The authors state (p.12) they have committed some questions due to low reliability score – the full details of the process need to be provided including which questions were omitted, which scale thy belonged to originally and what were the scores.

**Response:** We sincerely apologize for the erroneously entered statement in the initial manuscript, which has been removed from the revised version.

**Comment6:**The process by which the multivariate logistic regression model was developed is not well described, how were the original predictors chosen and how were some of them omitted and the rest retained?

**Response:** Thank you for your insightful reading and suggestion to write up the entire procedure that we followed while running the regression model. We also realized that it is beneficial to the readers. As a result, we attempted to show each step that we did take and highlighted it in the “Data analysis” section of the “Revised manuscript with Track Changes”, Page7, Line 200-206.

**Comment7:** The Hosmer-Lemeshow test of goodness of fit reported is usually not recommended due to low reliability. Providing a more fulsome description of the logistic regression conducted and the model fit indices is needed.

**Response:** Thank you for your thorough review and suggestion. There are, of course, numerous ways to report the goodness of fit test for a binary logistic regression model. In our case, almost all statisticians advised us to use a Hosmer-Lemeshow test of goodness of fit, which is why we included it in the initial manuscript. As you suggested, we ran additional tests for the goodness of model fit. The amended statements were highlighted in the “Data analysis” section of the “Revised manuscript with Track Changes”, Pages 7&8, lines 214-219. We would like to invite you to read the revised version of the data analysis section, where the majority of your comments and suggestions were discussed.

**Comment 8:** It is not clear why knowledge about safe-abortion services and the misconception about their cost are distinct factors – surely thinking a free service is unaffordable constitute an example for lack of knowledge – a better explanation as to why this were deemed distinct is needed.

**Response:** thank you for your insightful inquiry to discuss on the difference between the two variables. While writing the result, we made an error. Perceived affordability simply refers to the women's direct response that the cost of abortion services was affordable to them. As a result, we have changed the statement to "affordability of abortion services." The corrected statement was highlighted in the “Determinants of safe abortion service utilization” section of the “Revised manuscript with Track Changes”, Page11, Line 273-275. In addition, we also corrected the statement in the “Individual and health system-related characteristics” section of the “Revised manuscript with Track Changes”, Page9, Line 250.

Comment 9: The association between higher daily income and access to safe abortion services found is more perplexing than the discussion accounts for. Given the services are provided free of charge, is higher daily income a proxy for some other characteristic (e.g., higher executive functioning; greater autonomy in decision making)?

Response: Thank you for bringing this up. Despite the fact that abortion is free in Ethiopia, the extra expenses for transportation, food, and medication make it difficult for insecurely housed women to access a health facility for safe abortion services, which may lead them to prefer an unsafe abortion. We have tried to make the perplexing ideas more clear in the “Discussion” section of the “Revised manuscript with Track Changes”, Page9, Line 301-304 and Line 305-310.

In sum, I wish to reiterate that the manuscript is interesting and the study highly important and I hope some of my suggestions will prove helpful in revising this manuscript and resubmitting it for publication.

Response: We appreciate your in-depth review of our work, as well as your insightful comments and suggestions. We truly appreciate every point you raised as valuable input for our manuscript. Thank you very much for your endeavors.

**_____________________________________________________________________________________________________________END____________________________________**

**THANK YOU!!!**
